# Role of cooperative groups and funding source in clinical trials supporting guidelines for systemic therapy of breast cancer

**DOI:** 10.18632/oncotarget.24589

**Published:** 2018-02-28

**Authors:** Ariadna Tibau, Geòrgia Anguera, Fernando Andrés-Pretel, Arnoud J. Templeton, Bostjan Seruga, Agustí Barnadas, Eitan Amir, Alberto Ocana

**Affiliations:** ^1^ Oncology Department, Hospital de la Santa Creu i Sant Pau and Universitat Autònoma de Barcelona, Barcelona, Spain; ^2^ Research Foundation of the Paraplegics Hospital of Toledo, Toledo, Spain; ^3^ Department of Medical Oncology, St Claraspital, Basel and Faculty of Medicine, University of Basel, Basel, Switzerland; ^4^ Department of Medical Oncology, Institute of Oncology Ljubljana and University of Ljubljana, Ljubljana, Slovenia; ^5^ Division of Medical Oncology and Hematology, Department of Medicine, Princess Margaret Cancer Centre and the University of Toronto, Toronto, Ontario, Canada; ^6^ Translational Research Unit, Albacete University Hospital, Centro Regional de Investigaciones Biomédicas, Universidad de Castilla La Mancha, CIBERONC, Albacete, Spain

**Keywords:** funding source, sponsorship, cooperative groups, pharmaceutical industry, government or academic institutions

## Abstract

**Introduction:**

Clinical research is conducted by academia, cooperative groups (CGs) or pharmaceutical industry. Here, we evaluate the role of CGs and funding sources in the development of guidelines for breast cancer therapies.

**Results:**

We identified 94 studies. CGs were involved in 28 (30%) studies while industry either partially or fully sponsored 64 (68%) studies. The number of industry funded studies increased over time (from 0% in 1976 to 100% in 2014; p for trend = 0.048). Only 10 (11%) government or academic studies were identified. Studies conducted by GCs included a greater number of subjects (median 448 vs. 284; *p* = 0.015), were more common in the neo/adjuvant setting (*p* < 0.0001), and were more often randomized (*p* = 0.018) phase III (*p* < 0.0001) trials. Phase III trial remained significant predictor for CG-sponsored trials (OR 7.1 *p* = 0.004) in a multivariable analysis. Industry funding was associated with higher likelihood of positive outcomes favoring the sponsored experimental arm (*p* = 0.013) but this relationship was not seen for CG-sponsored trials (*p* = 0.53).

**Materials and Methods:**

ASCO, ESMO, and NCCN guidelines were searched to identify systemic anti-cancer therapies for early-stage and metastatic breast cancer. Trial characteristics and outcomes were collected. We identified sponsors and/or the funding source(s) and determined whether CGs, industry, or government or academic institutions were involved. Chi-square tests were used for comparison between studies.

**Conclusions:**

Industry funding is present in the majority of studies providing the basis for which recommendations about treatment of breast cancer are made. Industry funding, but not CG-based funding, was associated with higher likelihood of positive outcomes in clinical studies supporting guidelines for systemic therapy.

## INTRODUCTION

Clinical trials play a crucial role for the development of novel therapeutic agents in oncology [1]. Once a compound has been identified and assessed for activity in preclinical models, the evaluation in clinical studies for safety and clinical efficacy is mandatory. Indeed, the approval of a new agent should be based, ideally on an improvement in overall survival (or its valid surrogate), its quality, or both. Thus, well designed clinical trials need to be adequately funded and promoted. Clinical research of systemic therapy can be sponsored through non-profit organizations mainly public resources (i.e. government or academic institutions), by pharmaceutical companies (i.e. for profit), or both. Although the pharmaceutical industry plays a key role in the discovery of novel drugs, the collaboration with physicians in academic institutions is essential during clinical evaluation [2]. Therefore, studies can be run directly by private companies, or be supported independently by cooperative groups (CGs) or academic centres.

Within recent years, conduct of oncology clinical trials has become increasingly complex. This may be explained by different factors: more demanding regulatory requirements, diseases of great complexity, enhanced standards of care and increasing costs of clinical research. Recently, concerns have been raised by government funding agencies regarding a state of crisis conducting cancer clinical trials, particularly those run by CGs [3].

There are no data on the influence of CGs on outcome of breast cancer drug therapy trials. Here, we evaluate the impact of the funding source and role of CGs in the development of drugs for the treatment of breast cancer and explore the association of funding with the characteristics of these trials. We hypothesized that characteristics, outcome and the reported methodological quality of drug therapy trials for breast cancer differ depending on the funding source.

## RESULTS

### Selection strategy and characteristics of studies

**Figure 1:**
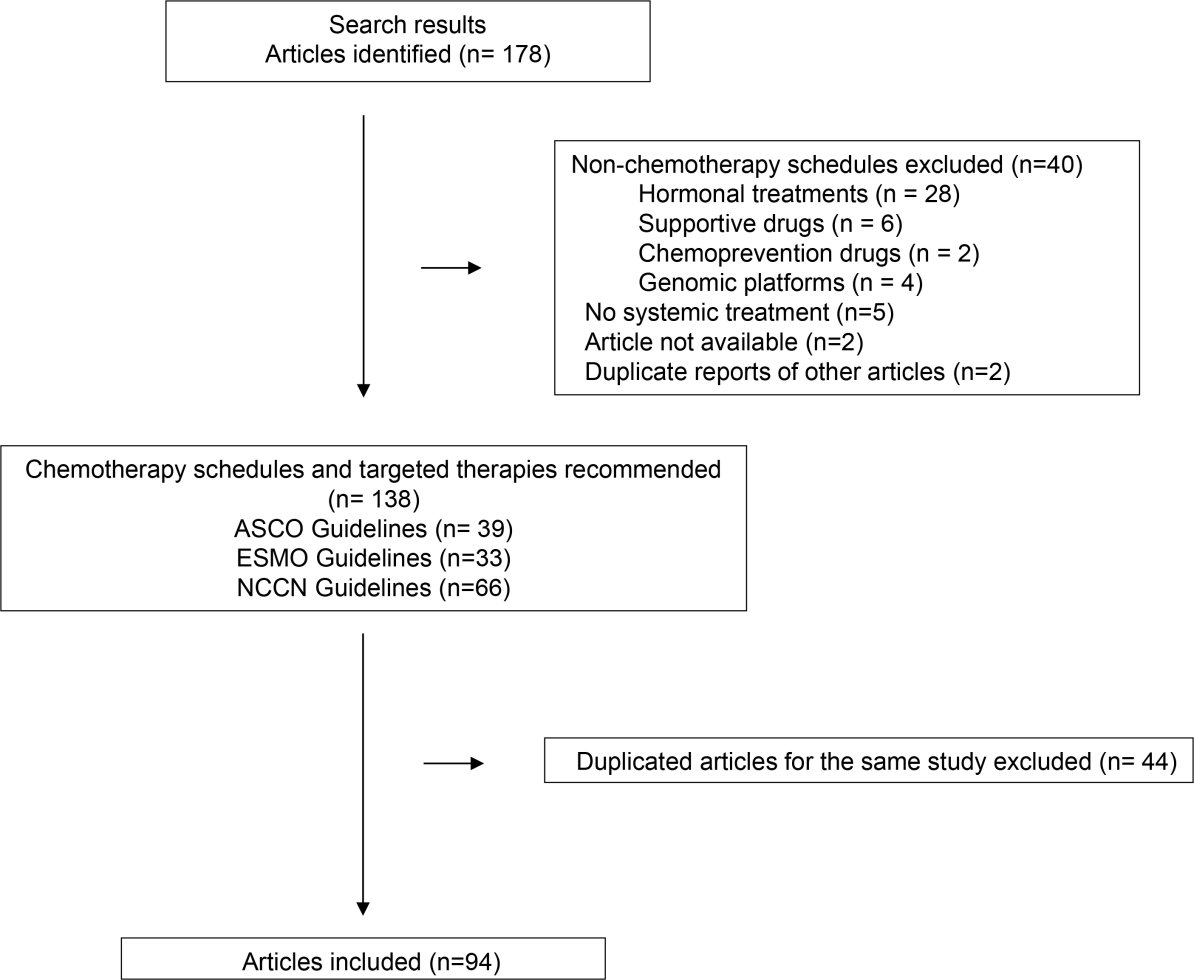
Flow chart showing search results

Figure 1 summarizes the article selection process. We identified 94 studies published between 1976 and 2014 supporting 112 approved and recommended cytotoxic agents and targeted therapies in different breast cancer settings.

Characteristics of the selected studies are shown in Table 1. The majority of these trials were multicenter (*n =* 79, 84%), randomized (*n =* 71, 75%), phase III (*n =* 56, 60%), performed in the metastatic setting (*n =* 71, 76%), and used intermediate end points (*n =* 83, 88%).

**Table 1:** Study characteristics

Characteristics	No. (%) 94
Sample Size, median (range)	657 (7–8381)
Year of publication	
1976–2003	30 (32%)
2004–2014	64 (68%)
Cooperative Groups	
Yes	28 (30%)
No	57 (61%)
Unspecified	9 (9%)
Industry funding	
Yes	64 (68%)
No	21 (22%)
Unspecified	9 (9%)
Type of agent	
Chemotherapy	61 (65%)
Chemotherapy and Targeted Agents	33 (35%)
Setting	
Neo/Adjuvant	23 (24%)
Metastatic	71 (76%)
Study design	
Randomized	71 (75%)
Single-arm	23 (25%)
Phase of study	
II	29 (31%)
III	56 (60%)
Retrospective trials	2 (2%)
No reported	7 (7%)
Blinding	
Yes	2 (2%)
No	69 (74%)
No applicable	23 (24%)
Primary endpoint	
Overall survival	11 (12%)
Intermediate endpoint	83 (88%)
Number of study centers	
Multiple	79 (84%)
Single	15 (16%)
Number of countries of study conduct	
Multiple	49 (52%)
Single	45 (48%)
Journal impact factors (IFs)	
Low (IF < 5.5)	11 (12%)
Intermediate (IF 5.5–18.5)	19 (20%)
High (IF > 18.5)	64 (68%)

### Funding sources of studies

**Figure 2:**
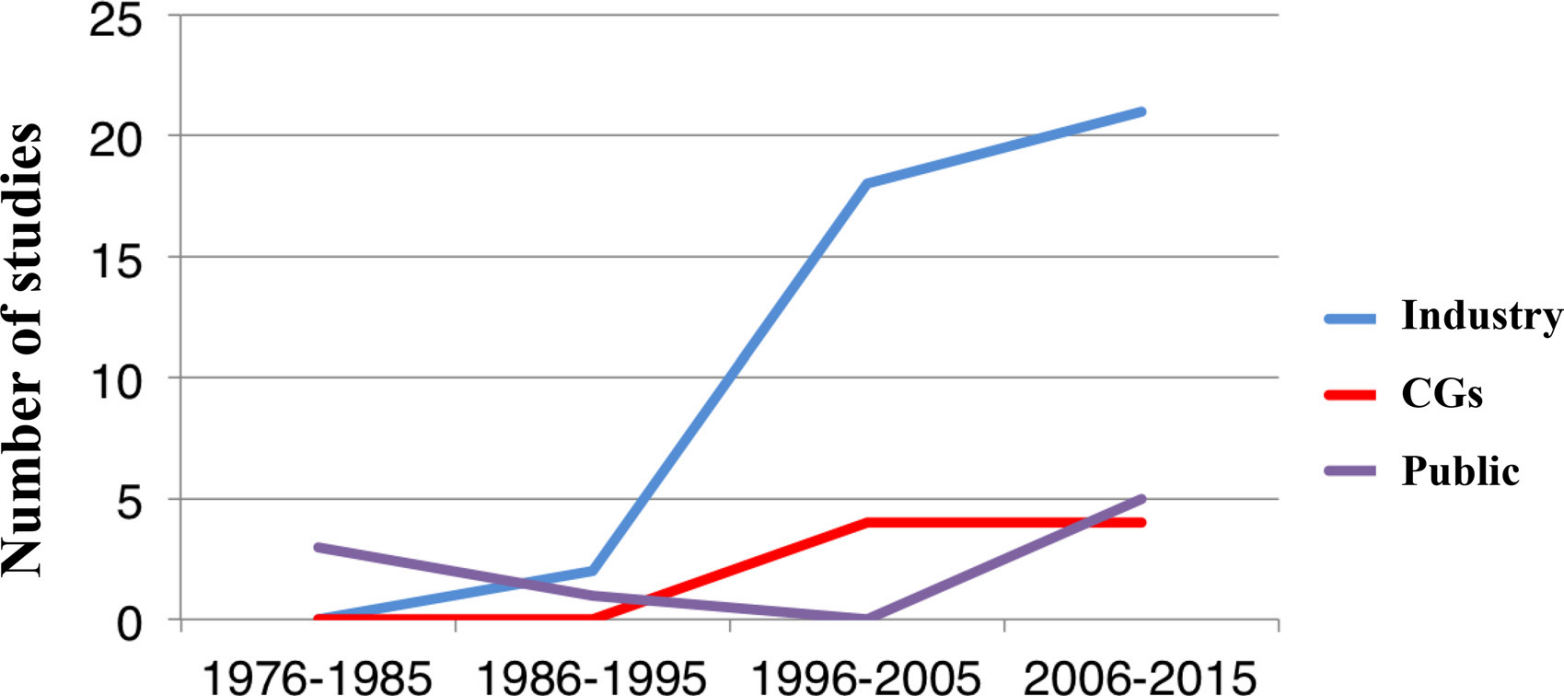
Role of funding source among clinical studies supporting guideline recommended systemic therapy for breast cancer over time

Cooperative Groups were involved in 28 (30%) studies. When evaluating industry-funded studies the number of studies increased to 64 studies (68%), being for 40 studies (42.5%) exclusively industry the funding source. Sixteen studies (57%) conducted by GCs were also funded by pharmaceutical industry. Government or academic studies were restricted to 10 (11%). In 9 cases (9%) funding source was not specified (Table 1). The number of studies sponsored by industry increased over time (from 0% in 1976 to 6% in 1995 and 100% in 2014, *p* for trend = 0.048) (Figure 2).

### Funding source and reporting quality of trials

Characteristics of clinical trials differed significantly according to the funding source (Table 2). Compared to industry funded studies, those supported by CGs included a greater number of subjects (median 448 vs. 284; *p =* 0.015), were more common in the neo/adjuvant setting (50% vs. 14%, *p <* 0.0001) and were more likely to be phase III (93% vs. 57%, *p <* 0.0001), randomized (93% vs. 70%, *p =* 0.018) and multicentre (100% vs. 84%, *p =* 0.027) studies. After adjusting for confounding factors, phase III trials remained significant predictors for CG-sponsored trials (OR 7.1 [95% CI 1,41–35,64] *p =* 0.004) in multivariable analysis.

**Table 2:** Clinical trials characteristics according to funding source

	*n* (%)	Cooperative Groups (%)	Non-Cooperative Groups (%)	*P* value
Number	85 (100%)	28 (40%)	57 (60%)	
Number of study subjects				0.015
Mean ± SD	670 ± 1248	1416 ± 2020	384.46 ± 493.94	
Median (range)	292 (22–8381)	448 (77–8381)	284 (28–3384)	
Number of study centres				0.027
Multiple	76 (89%)	28 (100%)	48 (84%)	
Single	9 (11%)	0 (0%)	9 (16%)	
Number of countries of study conduct				0.07
Multiple	48 (56%)	12 (43%)	36 (63%)	
Single	37 (44%)	16 (57%)	21 (37%)	
Blinding				1.0
Yes	2 (%)	1 (5%)	1 (2.5%)	
No	58 (%)	19 (95%)	39 (97.5%)	
Type of design				0.018
Randomized	66 (78%)	26 (93%)	40 (70%)	
Single Arm	19 (22%)	2 (7%)	17 (30%)	
Type of study				< 0.0001
Phase III	55 (69%)	25 (93%)	30 (57%)	
Phase II	25 (31%)	2 (7%)	23 (43%)	
Clinical setting				< 0.0001
Metastatic	63 (74%)	14 (50%)	49 (86%)	
Neo/adjuvant	22 (26%)	14 (50%)	8 (14%)	
Use of drug combinations				0.27
Yes	57 (67%)	21 (75%)	36 (63%)	
No	28 (33%)	7 (25%)	21 (37%)	
Primary Endpoint				0.42
Overall Survival	7 (8%)	1 (4%)	6 (10.5%)	
Intermediate Endpoint	78 (92%)	27 (96%)	51 (89.5%)	
Journal Impact Factor (IF)				0.92
Low (IF < 5.5)	7 (8%)	2 (7%)	5 (9%)	
Intermediate (IF 5.5–18.5)	16 (18%)	6 (21%)	10 (18%)	
High (IF > 18.5)	62 (74%)	20 (72%)	42 (73%)	

### Association of funding source with the outcome

All RCT could be assessed for efficacy outcomes. Industry funding was associated with higher likelihood of positive outcomes supporting guideline recommended systemic therapy for breast cancer (61% for not industry-sponsored vs. 88% for industry-sponsored, *p =* 0.013). In contrast, an association between CG funding and positive study outcome was not found (78% CG-sponsored vs. 84% not-sponsored by CGs, *p =* 0.53).

## DISCUSSION

In the present study we explore the role of CGs and academic research and the funding source of clinical studies supporting guidelines for systemic therapy in breast cancer. Of note, pharmaceutical industry involvement is present in the majority of clinical studies including those run by CGs. We observed that such funding has increased over time. In addition, the role of CGs has proportionally decreased although a presence is still observed in the adjuvant setting. Government or academic research is very limited in breast cancer.

Our article has relevant clinical implications as it describes the trend in clinical research in this tumor type and how sponsorship has changed from being largely academic to largely commercial. The fact that exclusively academic research is a limited contributor to guideline recommendations highlights the limitations to develop relevant hypotheses when they come from academic centers and do not have a potential commercial interest. Some clinical problems are not linked with a potential profit and therefore are not attractive targets for industry funded research. In addition to this, clinical research has become more expensive and complex what clearly justifies the small number of government or academic studies. Conversely, public funding is not sufficient to cover all costs associated with independent clinical research. In this context, it is unknown how the decrease in independent research can affect the resolution of clinical problems observed by independent investigators.

In this study, a positive outcome favoring the experimental arm was more likely for industry funded trials than for CGs-sponsored trials. In line with this is the fact that pharmaceutical industry funding and support of biomedical research has dramatically increased over the last years being now the main funding source [4, 5]. This has led to concerns regarding a potential source of bias due to a link between industry funding and an increased likelihood of pro-industry results and conclusions [6–8] although methodological quality has been shown to be similar in trials with industry collaboration than those without industry funding [9]. How to solve this problem is not easy, but mechanisms to decrease complexity at least from regulatory bodies could permit to perform less expensive studies that could be funded totally with public sources.

Of interest, the fact that the role of CGs has proportionally decreased over time indirectly measures the influence of industry in independent research and the complexity to execute multinational studies. Although the primary focus of CGs is perform large, definitive, randomized phase III studies, they have also played a role in the development of novel therapeutic drugs in phase I and phase II trials [10]. However, CGs still have a role in areas where the inclusion of a high number of patients are necessary like in the adjuvant setting. As expected, our results show that studies conducted by GCs were more often phase III trials and suggesting that well-designed phase III RCTs can prevent bias in the comparison of treatments and provide a sound basis for changes in clinical practice [1]. In this area a large number of studies were sponsored by CGs with funding from pharmaceutical companies.

Our study has limitations. Funding data and role of the sponsors was not always adequately reported. In our study funding data abstraction relied on subjective interpretations of language. To address this limitation, we confirmed doubtful cases with a third author. As our study was limited to breast cancer, it is unclear whether our results could be generalizable to other cancer sites. However, for tumors with a similar incidence the probability to observe similar findings is high.

In conclusion, we evaluated the role of CGs in breast cancer and its funding source, identifying a substantial presence of those funded by the pharmaceutical industry, with a decrease in the proportion of those supported by CGs exclusively. Endorsement of experimental drugs was more frequent for studies with industry funding.

## MATERIALS AND METHODS

### Search strategy

The most recent versions of the American Society of Clinical Oncology (ASCO) [11], European Society of Medical Oncology (ESMO) [12], and National Comprehensive Cancer Network (NCCN) guidelines [13] were searched to identify chemotherapy drugs and targeted therapies endorsed to treat early-stage and metastatic breast cancer. Subsequently, we identified original articles published between 1976 and 2014 that supported those drug recommendations. When multiple (or overlapping) articles for the same study were identified, the most recent publication was included. Articles regarding genetic risk evaluation, screening, type of surgery, hormonal therapy, and radiotherapy were excluded. Two reviewers (G.A. and A.T.) independently assessed each trial for the funding source, study characteristics, and outcomes. Disagreements were resolved by a third author (AO).

### Data extraction

Data on the journal and year of publication, total number of study subjects, site locations (number of study countries and number of study centres), type of study drug, phase of the study (II vs. III), the design of the study (randomized vs. single-arm), and intervention arms were recorded. The 2015 journal impact factors (IFs) were obtained from Thomson Reuters Journal Citation Reports and classified as low (IF < 5.5), intermediate (IF 5.5–18.5), or high (IF > 18.5).

### Funding information and outcome assessment

We also extracted how funding sources and sponsorship were defined. Funding sources were classified as CGs, industry or government or academic organizations based on the recorded lead sponsor and/or collaborator and/or funding information reported in the disclosure in the published manuscript. “Lead sponsor” was defined as the organization responsible for overseeing the trial and analyzing study data, while “collaborator” was defined as an organization other than the sponsor that provides support for a clinical study, including funding, design, implementation, data analysis, or reporting [14]. If a CG was listed as a lead sponsor or as a collaborator the trial was considered “CG-sponsored”. If industry was the lead sponsor or was listed as a collaborator, the trial was classified as “industry sponsored”. Of the CG-sponsored trials, those with full or partial funding from industry were categorized as “industry funded”. Trials sponsored only with public funding (e.g. such as National Cancer Institute [NCI] and National Institutes of Health [NIH] or academic institutions) as “government or academic funded”. When no funding source was declared, these trials were classified as unspecified.

A positive outcome was defined as the pre-specified measure of success for the primary outcome has been met -that is, whether a *P* value of less than 0.05 has been achieved for the difference in treatments [15]. Of note, randomized controlled trials (RCTs) with a statistically significant result favoring experimental drug for the primary outcome or at least one dose showing significant efficacy in primary outcome without increased adverse events, were considered as positive clinical trials. RCTs with a statistically significant result in favor of the active comparator drug over experimental drug or when significant safety concerns were found for primary outcome were considered negative clinical trials. Outcome could not be assessed if no study intervention arm was declared as experimental a priori.

### Statistical analysis

Means and proportions were used to describe the data. Comparisons of proportions between groups were conducted using chi-square test. Means comparisons were assessed using Student’s *t* test or Mann-Whiteny’s U, as appropriate. After categorization of the years during which the studies were conducted we analyzed trends over time using the Cochran-Armitage test. Multivariable analysis was conducted using binary logistic regression with CG as dependent variable and studied factors as independent and Conditional Backstep as variable selection method, with an inclusion criteria of 0.05 and an exit criteria of 0.10. All *p* values were two-tailed. Logistic regression was used to adjust for potential confounders when assessing the association between the funding source and study outcome. Data analyses were conducted using SPSS version 22.

## References

[1] Tannock IF, Amir E, Booth CM, Niraula S, Ocana A, Seruga B, Templeton AJ, Vera-Badillo F (2016). Relevance of randomised controlled trials in oncology. Lancet Oncol.

[2] Bodenheimer T (2000). Uneasy alliance--clinical investigators and the pharmaceutical industry. N Engl J Med.

[3] Kola I, Landis J (2004). Can the pharmaceutical industry reduce attrition rates?. Nat Rev Drug Discov.

[4] Booth CM, Cescon DW, Wang L, Tannock IF, Krzyzanowska MK (2008). Evolution of the randomized controlled trial in oncology over three decades. J Clin Oncol.

[5] Bekelman JE, Li Y, Gross CP (2003). Scope and impact of financial conflicts of interest in biomedical research: a systematic review. JAMA.

[6] Tibau A, Bedard PL, Srikanthan A, Ethier JL, Vera-Badillo FE, Templeton AJ, Ocaña A, Seruga B, Barnadas A, Amir E (2015). Author financial conflicts of interest, industry funding, and clinical practice guidelines for anticancer drugs. J Clin Oncol.

[7] Djulbegovic B, Lacevic M, Cantor A, Fields KK, Bennett CL, Adams JR, Kuderer NM, Lyman GH (2000). The uncertainty principle and industry-sponsored research. Lancet.

[8] Stelfox HT, Chua G, O’Rourke K, Detsky AS (1998). Conflict of interest in the debate over calcium-channel antagonists. N Engl J Med.

[9] Linker A, Yang A, Roper N, Whitaker E, Korenstein D (2017). Impact of industry collaboration on randomised controlled trials in oncology. Eur J Cancer.

[10] Mauer AM, Rich ES, Schilsky RL (2007). The role of cooperative groups in cancer clinical trials. Cancer Treat Res.

[11] http://ascopubs.org/jco/site/misc/specialarticles.xhtml

[12] http://www.esmo.org/Guidelines/Breast-Cancer

[13] https://www.nccn.org/store/login/login.aspx?ReturnURL=https://www.nccn.org/professionals/physician_gls/pdf/breast.pdf

[14] Califf RM, Zarin DA, Kramer JM, Sherman RE, Aberle LH, Tasneem A http://ClinicalTrials.gov.

[15] Pocock SJ, Stone GW (2016). The Primary Outcome Is Positive - Is That Good Enough?. N Engl J Med.

